# Evidence for a lineage of virulent bacteriophages that target *Campylobacter*

**DOI:** 10.1186/1471-2164-11-214

**Published:** 2010-03-30

**Authors:** Andrew R Timms, Joanna Cambray-Young, Andrew E Scott, Nicola K Petty, Phillippa L Connerton, Louise Clarke, Kathy Seeger, Mike Quail, Nicola Cummings, Duncan J Maskell, Nicholas R Thomson, Ian F Connerton

**Affiliations:** 1 Division of Food Sciences, School of Biosciences, University of Nottingham, Sutton Bonington Campus, Loughborough, Leicestershire, LE12 5RD, UK; 2 Department of Veterinary Medicine, University of Cambridge, Cambridge, CB3 0ES, UK; 3 Wellcome Trust Sanger Institute, Wellcome Trust Genome Campus, Hinxton, Cambridge, CB10 1SA, UK; 4 Defence Science and Technology Laboratory, Porton Down, Salisbury, SP4 0JQ, UK

## Abstract

**Background:**

Our understanding of the dynamics of genome stability *versus *gene flux within bacteriophage lineages is limited. Recently, there has been a renewed interest in the use of bacteriophages as 'therapeutic' agents; a prerequisite for their use in such therapies is a thorough understanding of their genetic complement, genome stability and their ecology to avoid the dissemination or mobilisation of phage or bacterial virulence and toxin genes. *Campylobacter*, a food-borne pathogen, is one of the organisms for which the use of bacteriophage is being considered to reduce human exposure to this organism.

**Results:**

Sequencing and genome analysis was performed for two *Campylobacter *bacteriophages. The genomes were extremely similar at the nucleotide level (≥ 96%) with most differences accounted for by novel insertion sequences, DNA methylases and an approximately 10 kb contiguous region of metabolic genes that were dissimilar at the sequence level but similar in gene function between the two phages. Both bacteriophages contained a large number of radical *S*-adenosylmethionine (SAM) genes, presumably involved in boosting host metabolism during infection, as well as evidence that many genes had been acquired from a wide range of bacterial species. Further bacteriophages, from the UK *Campylobacter *typing set, were screened for the presence of bacteriophage structural genes, DNA methylases, mobile genetic elements and regulatory genes identified from the genome sequences. The results indicate that many of these bacteriophages are related, with 10 out of 15 showing some relationship to the sequenced genomes.

**Conclusions:**

Two large virulent *Campylobacter *bacteriophages were found to show very high levels of sequence conservation despite separation in time and place of isolation. The bacteriophages show adaptations to their host and possess genes that may enhance *Campylobacter *metabolism, potentially advantaging both the bacteriophage and its host. Genetic conservation has been shown to extend to other *Campylobacter *bacteriophages, forming a highly conserved lineage of bacteriophages that predate upon campylobacters and indicating that highly adapted bacteriophage genomes can be stable over prolonged periods of time.

## Background

Bacteriophages (phages) are naturally occurring predators of bacteria that are ubiquitous in the environment; they are almost certainly the most abundant biological entities on the planet [1]. It is commonly accepted that phage genomes are an extremely rich source of novel and unique DNA sequences, and that phage genomes are highly variable, often showing a mosaic patchwork of genetic segments acquired from a range of sources including other phages and bacteria. However, this view is almost certainly an oversimplification; as more phage genomes become available it is clear that closely related phage lineages exist in the environment which may be stable over appreciable time-scales and geographic areas [2,3]. Understanding how these lineages evolve and adapt to their hosts will provide useful insights into the phage pan-genome, genetic flux within communities and phage genome stability.

The general availability of phages in the environment coupled with their ease of isolation and cultivation has led them to be used in a variety of ways. Phages have been instrumental in the development of molecular biology, their use as typing tools for bacterial pathogens continues today and recently there has been a resurgence of interest in phage intervention to control pathogens, so called 'bacteriophage therapy'. The closely related zoonotic pathogens *Campylobacter jejuni *and *Campylobacter coli *are major causes of infectious bacterial gastroenteritis in humans [4-6], and have been associated with rare but serious, sometimes fatal, neurological sequelae such as Guillain-Barré syndrome, Miller-Fisher syndrome and the onset of reactive arthritis [7-9]. Using phages to reduce *Campylobacter *at multiple stages of the food chain is a promising sustainable intervention strategy but requires detailed knowledge of phage genomes at the sequence level. Although previous studies have shown that the application of phages can effectively reduce *Campylobacter *contamination [10-12], to date the most common use of *Campylobacter *phages has been in typing schemes allowing the discrimination between different *Campylobacter *isolates [13,14]. Previous studies have shown that all the *Campylobacter *phages examined to date belong to either the *Myoviridae *or *Siphoviridae *tailed phage families [15]. Based on genome size, they have been categorized into three groups; group III 130 - 140 kb, group II 180 - 190 kb and the typing phages NCTC 12676 and NCTC 12677, which are reported to be ~320 kb in size, as group I [16,17]. Molecular characterization of these phages has been slow, with many of the currently available *Campylobacter *phages being extremely refractory to genomic analysis. In many cases phage genomic DNA is resistant to digestion with any of the standard restriction endonucleases, although, *Hha*I has proven to be useful to discriminate some group III phages [16-18]. However, thorough characterization of *Campylobacter *specific phages (indeed any phage intended for therapeutic applications) is a prerequisite to avoid the inadvertent transfer or mobilization of harmful genes [19].

In this work we report the first full genome sequences, analysis of virion proteins and the genome analysis for two large virulent *Campylobacter *specific phages; CP220 a phage isolated from chickens in 2003 [10,20] and CPt10 a member of the UK *Campylobacter *typing scheme phages, isolated from a slaughterhouse environment prior to 1989 [21]. Both display typical genome sizes for group II phages, a morphology typical of the *Myoviridae *phages [17,20] and exhibit broad but different host ranges with both phages notably able to lyse *Campylobacter jejuni *and *Campylobacter coli *isolates. The genome sequences illuminate a very highly conserved *Campylobacter *specific phage lineage that has survived the ongoing competition between host and virus.

## Results and Discussion

### Genome structure

**Figure 1:**
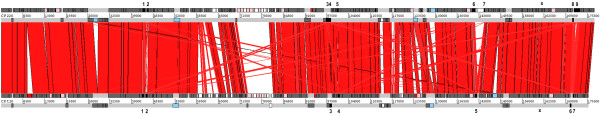
**Genome alignments of CP220 and CPt10**. Nucleotide alignment of the virulent bacteriophages CP220 (top) and CPt10 (bottom) generated using the Artemis Comparison Tool (ACT). The CDS regions are indicated by bars top and bottom showing the 'forward' and 'reverse' strands. CDS regions are coloured according to the following key: grey - areas of nucleotide identity, white - significantly different CDS regions present in only one of the genomes, solid black - repeat regions (numbered according to the current genome annotation), red - DNA modification proteins, blue - insertion elements, pink - radical SAM proteins. Red bars indicate regions of sequence homology, the diagonal lines link multiple repeat regions in both genomes indicating the conserved nature of these sequences throughout both genomes.

Two virulent *Campylobacter *phages have been sequenced to reveal double stranded DNA genomes of 177 493 bp (CP220) and 175 720 bp (CPt10), a comparison of the genomes can be found in Table 1. The full annotated genome sequences have been deposited with the EMBL nucleotide sequence database with accession numbers; CP220 [EMBL: FN667788] and CPt10 [EMBL: FN667789]. These phages have a GC content comparable to that found in the host species, 30.5%, 30.3% and 31.1% for *C. jejuni *NCTC 11168, RM1221 and *C. coli *RM2228 respectively [22]. Both the CP220 and CPt10 genomes show a distinct strand bias for putative protein coding sequences (CDS), (see Additional file 1 - Table S1) with the majority of the CDSs lying on the 'forward' strand, an observation in accordance with that for many other phages where a similar bias in orientation is observed [23,24]. In many bacterial species predominant gene orientation is frequently parallel to the direction of replication of the forward strand, thus minimising interference between RNA transcription and DNA replication. However, both phage genomes contain notable exceptions to this strand bias, including two distinct sections of DNA where contiguous runs of CDSs lie on the reverse strand (Figure 1).

**Table 1:** Comparison of basic parameters for CP220 and CPt10 phage genomes

	Genome size bp^a ^(+/- variable repeats)	Genome % G+C content	CDS % G+C content	Number of CDS^b^	Coding Density^b ^%	Strand Bias 'forward'
CP220	177 493/171 841	27.4	28.2	194	88.4	173/194

CPt10	175 720/173 299	27.3	28.0	201	89.7	180/201

^a ^Genome sizes are given both for the sequences as currently assembled and excluding repeat regions which may be variable in size.

^b ^The number of CDS regions and the coding density is based on the proportion of the genome covered by discrete coding regions. It does not account for CDS regions produced from internal initiation sites or alternative gene products produced by splicing, post-translational processing or other mechanisms that may increase the number of protein products produced from the genome.

In most cases the strand alignment of the open reading frames is supported by the apparent change in strand AG content, with a distinct preference for A and G bases in the coding strand. This bias was also reflected in the codon usage [25]. Comparison of the codon usage frequencies from the entire genome sequences of CP220 and CPt10 and from several *C. jejuni *and *C. coli *genome sequences reveals that in cases where synonymous codons are available, the phages show a preference for alternative codons to those employed by campylobacters (Additional file 2). There is a small but definite bias towards codons with A or U at the third position, which probably reflects the high A+T content of these phage genomes, although as noted above the phage genomes closely match that of host campylobacters in terms of their overall base composition.

The phages carry tandem tRNA genes with arginine and tyrosine type anticodons. Unusually, the Tyr-tRNA in CP220 shows a single base substitution in a highly conserved tRNA residue but it is not clear how this base change would impact on function. Examination of the phage codon usage shows that the two codons recognized by these tRNAs are represented more frequently in the phage coding regions than in *Campylobacter*, and thus probably explains the evolutionary pressure for their retention. What is perhaps more surprising is that these phages don't carry more tRNA genes, for example T4 encodes eight tRNAs [26], as there are a number of other codons that are represented more frequently in phage CDSs than in *Campylobacter*. The codon preferences observed could be an evolutionary relic, considering the wide range of organisms that appear to have contributed to the phage genomes, or it may be a strategy to fully utilize the available tRNA pools in *Campylobacter *to maintain key host functions whilst translating phage proteins necessary for replication and reproduction.

Of the 201 and 194 predicted CDSs identified for CPt10 and CP220 respectively, some show significant conservation (greater than 20% identity at the protein level) to a range of structural and enzymatic proteins from phage T4 (Table 2). Proteins Gp20 (portal vertex protein) and Gp23 (the major capsid protein) are both represented in the *Campylobacter *phage sequences with identity to the corresponding T4 proteins of 28% and 31% respectively. The observation of sequence conservation suggests that these phages belong to the diverse T4-like phage superfamily but somewhat distant to the archetypal member of the group. The overall architecture of the genome appears to follow a modular construction, with most of the phage structural and DNA replication genes clustered in the left and right arms of the genomes as represented in Figure 1, while the central third of the genome comprises a much more heterogeneous selection of genes involved with metabolic processes. However, in common with other phage genome sequences, a large proportion of putative CDSs 85/194 from CP220 and 95/201 from CPt10 show no significant matches to sequences currently deposited in databases.

**Table 2:** Comparison of T4 and CP220 Structural Proteins

CP220 CDS	T4 Length^a^	CP220 Length^a^	T4 Gene Function^b^	CP220/T4 Identity (%) E value^c^
CPT_0001	610	744	Gp17 Terminase subunit with nuclease and ATPase activity; binds single-stranded DNA, Gp16 and Gp20	143/436 (32%) E = 4e^-47^

CPT_0005	516	503	Gp39 DNA topoisomerase large subunit	186/516 (35%) E = 4e^-73^

CPT_0009	342	347	Gp61 DNA primase subunit	73/330 (22%) 0.017

CPT_0010	319	304	Gp44 Clamp loader subunit, DNA polymerase accessory protein	95/319 (29%) E = 8e^-27^

CPT_0011	305	352	RNaseH ribonuclease	56/151 (37%) E = 6e^-16^

CPT_0029	157	178	Gp49 EndoVII packaging and recombination endonuclease VII	28/108 (25%) E = 0.11

CPT_0030	524	569	Gp20 Portal vertex protein of head	130/456 (28%) E = 5e^-40^

CPT_0033	487	429	Gp30 DNA ligase	128/484 (26%) E = 2e^-23^

CPT_0034	659	516	Gp18 Tail sheath monomer	58/235 (24%) E = 3e^-04^

CPT_0037	134	120	Gp25 Baseplate wedge subunit	26/66 (39%) E = 2e^-07^

CPT_0041	660	1214	Gp6 Baseplate wedge subunit	86/389 (22%) E = 0.025

CPT_0045	163	252	Gp19 Tail tube protein	49/176 (27%) E = 9e^-11^

CPT_0046	150	158	Gp4 Head completion protein	44/147 (29%) E = 5e^-08^

CPT_0048	185	255	Gp55 Sigma factor for T4 late transcription	33/123 (26%) E = 0.37

CPT_0051	521	444	Gp23 Major head protein	131/412 (31%) E = 2e^-41^

CPT_0053	659	578	Gp18 Tail sheath protein	149/436 (34%) E = 1e^-51^

CPT_0058	163	196	Gp19 Tail tube protein	41/170 (24%) E = 5e^-09^

CPT_0115	898	882	Gp43 DNA polymerase	244/901 (27%) E = 4e^-56^

CPT_0125	475	445	Gp41 DNA primase-helicase subunit	120/453 (26%) E = 1e^-34^

CPT_0148	587	472	UvsW RNA-DNA and DNA-DNA helicase, ATPase	124/407 (30%) E = 5e^-42^

CPT_0174	272	456	Gp15 Tail sheath stabilizer and completion protein	54/214 (25%) E = 1e^-05^

CPT_0177	301	306	Gp32 Single-stranded DNA binding protein	65/212 (30%) E = 6e^-12^

CPT_0181	374	361	RnlA RNA ligase 1 and tail fiber attachment catalyst	80/297 (26%) E = 3e^-10^

CPT_0193	212	236	Gp21 Prohead core protein protease	52/153 (33%) E = 2e^-08^

^a ^Length refers to the number of amino-acid residues in the CDS and are calculated from the genomic sequence

^b ^T4 gene product and functions are according to Miller [26]

^c ^Identity and E values are from NCBI BLAST results, performed on 5^th ^January 2009

Direct comparison of the two genomes reveals an extremely high level of conservation (Figure 1), with an average nucleotide identity over the entire length of the phage chromosomes of 96.2%. So high is the conservation between these two phages it is possible to identify the boundaries of insertion or deletion events encompassing discrete CDSs. Comparison of the genomes revealed that 26 CDSs are unique to CP220 and 28 are unique to CPt10. The majority of these have no significant matches to database sequences, however, two of the unique sequences in CPt10 code for putative DNA methyltranferases not present in CP220. CPt10_0091 shares 59% identity (over 588 amino acids) with a type III restriction/modification methyltranferase subunit from *Campylobacter jejuni*. Although the gene has a confirmed frame-shift mutation in a poly-adenosine tract towards the distal end of the gene, the full-length product could still be expressed through a process of transcriptional slippage [27,28]. The second putative methyltranferase, CPt10_1471, shows similarity to DNA methyltranferases from both *Clostridium *and *Campylobacter *sp. CP220 and CPt10 have different *Campylobacter *host ranges (data not shown), the two identified methyltranferases may enable CPt10 to avoid host restriction defences in some strains through modification of its own DNA. In addition methylation is known to be involved in control of gene expression in many organisms and these enzymes may be involved in such a process during CPt10 infection, either through modification of its own DNA or by modification and possibly silencing of host gene expression.

The largest region differentiating these two phages is a cluster encoding 10 CDSs in CPt10, and 12 in CP220. The two clusters show little relationship at the nucleotide or amino-acid sequence level but show remarkable functional conservation, where five CDSs from each cluster possess conserved radical *S*-adenosylmethionine (SAM) domains and each cluster appears to posses a putative glycine amidinotransferase. These regions are unlikely to have diverged from a common progenitor as this would require localised mutation rates in these regions to be much higher than in the surrounding phage genome. It is more likely these sequences have been acquired *en masse *from a related organism, whether phage or bacterial, by homologous or non-homologous recombination.

In total there are 11 proteins belonging to the radical SAM superfamily in CP220 and 12 in CPt10. Radical SAM proteins are involved in the cleavage of unreactive C--H bonds in a range of biochemical processes as diverse as DNA repair, lysine metabolism and the generation of vitamins and cofactors such as biotin, heme and thiamine [29]. The presence of so many radical SAM proteins in these phage genomes is highly unusual. Approximately 27,000 proteins have so far been assigned to this superfamily, of these the overwhelming majority (~22,800) are bacterial proteins, with only around 22 having been identified in phage genomes thus far [30]. These proteins are associated with oxygen-independent oxidation reactions and are often found in anaerobic organisms; presumably they may also confer a metabolic advantage at low oxygen tensions, precisely the conditions that *Campylobacter *faces in the gut lumen. This analysis identifies a central portion of both phages, carrying five radical SAM genes, as being an interchangeable module with related functions that are likely to be non-essential for phage proliferation. However, it is possible that these metabolic enzymes could temporarily improve the competitive fitness of infected *Campylobacter *thus also benefiting the phage.

### Repeat regions and genome expansion

**Figure 2:**
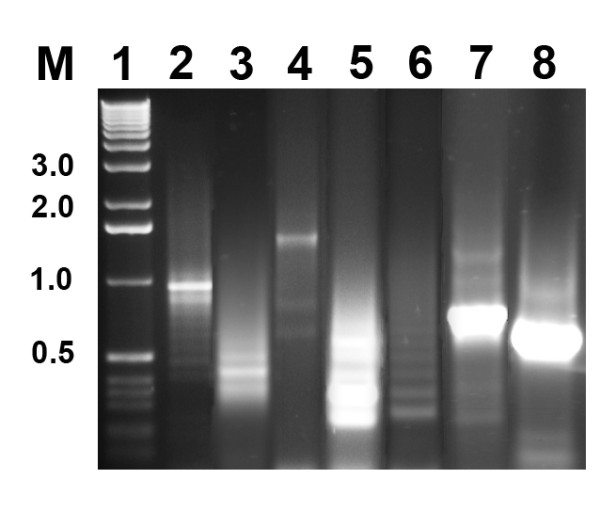
**Repeat regions present in CP220**. PCR amplification of repeat regions from CP220 showing the range of products produced from a single population. Lanes; M - size in kbp, 1 - size marker (1 kb DNA ladder, Invitrogen, Paisley, UK), 2 - repetitive region (RR) as currently annotated in the CP220 genome RR1, 3 - RR3/4, 4 - RR6, 5 - RR8, 6 - RR9, 7 - RRx, 8 - RR7.

CP220 and CPt10 both carry regions of repetitive DNA, accounting for 3.2% (at 10 loci) and 1.4% (at 8 loci) of their respective genomes. In all but one instance, common to both phages, the repeat regions are extragenic. The sole exception being a 21 bp repeat motif found within the putative coding sequences CPT_0180 and CPt10_1891 in CP220 and CPt10 respectively. The remaining repetitive regions contain between 3 and 17 copies of an approximately 75 bp core repeat unit (Additional file 1 - Table S2), with minor sequence polymorphisms evident between units both within and between repeat regions. PCR was used to further examine these repeats in CP220. Six out of the seven regions examined were shown to produce amplicons of mixed lengths, despite the template originating from a single plaque purified population of CP220 (Figure 2). Thus, the length of these regions would appear to be subject to variation during phage proliferation and may serve to create genome diversity. Examination of individual repeat regions shows the presence of tandem repeats containing the same sequence polymorphisms, suggesting that expansion (or contraction) could occur by slip-strand mispairing during DNA replication. Other repeat regions show evidence that recombination or translocation of repeat units has occurred, for example sequences more characteristic of repeat units within regions 6 and 8 are present in repeat region 9 of CP220, so either identical sequence changes have occurred at multiple sites or more likely the repeat units have propagated themselves in the phage genome.

The generation of genomes of variable length could be an adaptive mechanism of the phages in response to conditions in their host or environment, perhaps by influencing the packaged genome and gene dosage in the terminally redundant regions. However, it is difficult to see to what extent these relatively small changes in genome size would contribute to such a mechanism. More likely, they may serve as recombination substrates allowing reassortment of phage genes. For example, there are three instances in CP220 (flanking CPT_0046, CPT_0110 to CPT_0113 and CPT_0159 to CPT_0160), where one or more discrete CDSs are bordered by repeat sequences. The relative positions of the repeat regions within the CP220 and CPt10 genomes are generally conserved (Figure 1). Thus, they could also facilitate the exchange of larger segments between closely related phages. It is clear however that any CP220 and by corollary CPt10 recovered, is actually but a single representative of a larger family of related genomes under continual size-flux, the relative proportions of which may be dictated by selective environmental pressures.

### Tail proteins

**Figure 3:**
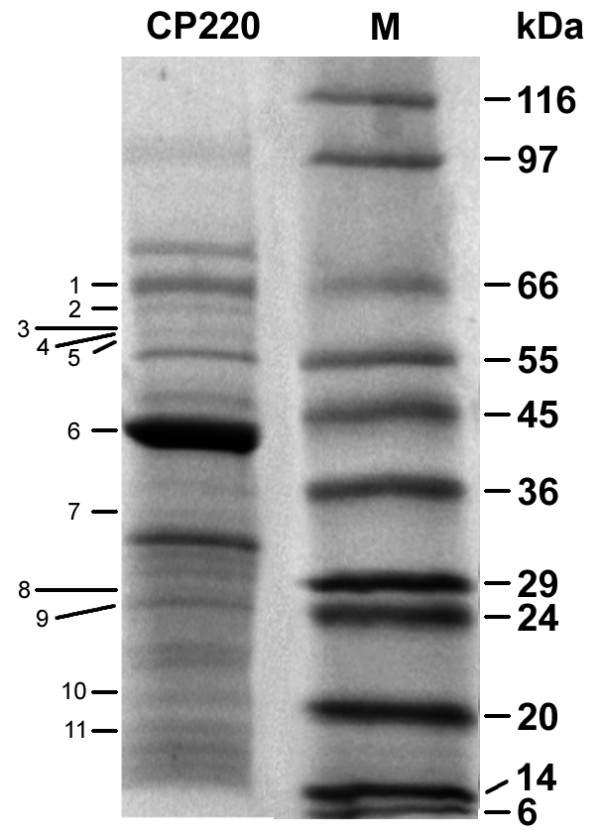
**Virion proteins from CP220**. SDS-PAGE analysis of CP220 phage virion proteins. M - molecular size markers. Numbers refer to protein sequences excised from the gel along with the equivalent genome CDS designation and description in T4 if known (the protein molecular masses correspond with the translation products of the nucleotide sequence, kDa); 1 - CPT_0030 - Gp20 portal vertex protein of head (66.3), 2 - CPT_0053 - Gp18 tail sheath (64.2), 3 - CPT_0034 - Gp18 tail sheath (59.2), 4 - CPT_0052 - Gp18 tail sheath (58.4), 5 - CPT_0103 - unknown (57.2), 6 - CPT_0051 - Gp23 major capsid (48.6), 7 - CPT_0186 - Gp48 base plate tail tube cap (34), 8 - CPT_0175 - Gp19 tail tube (27.4), 9 - CPT_0118 - unknown, neck protein (26.9), 10 - CPT_0045 - Gp19 tail tube (28.7), 11 - CPT_0046 - Gp4 head completion (18.6).

Both CP220 and CPt10 have multiple genes whose products show homology to Gp18 - the tail sheath and Gp19 - tail tube proteins. Protein sequencing of tryptic fragments of proteins excised from SDS-PAGE demonstrates that at least two types of Gp18 and two types of Gp19 are present in mature CP220 virions (Figure 3). It is unknown whether the individual protein types are mixed within a single phage virion or if discrete populations exist, with each mature virion containing a single protein type. Regardless, these variants arise within virus populations originally propagated from single plaques. The versions of Gp18 are associated with mobile elements; this suggests a mechanism whereby copies of the tail sheath genes may be exchanged, thereby enabling reassortment between related phages and possibly modulating their host range.

### Phage replication and packaging

The phages carry genes involved in replication including; putative genes for DNA primase, sliding clamp, sliding clamp loader proteins, DNA polymerase, RNaseH and DNA ligase, thus replication of the phages is most likely independent of the host replication complex. Several putative transcription factors are present and may coordinate gene expression throughout the phages lifecycle; these genes provide inviting targets to examine transcriptional control during the infection process in these phages.

Putative terminases with significant similarity to the T4 large terminase subunit Gp17, 32% identity (143/436 amino acids) for CP220, have been identified in both phages. Interestingly, the CP220 protein exhibits sequence conservation within the C-terminal domain, responsible for the DNA nuclease activity, but not the N-terminus which has an ATPase function and is responsible for DNA binding [31]. CP220 also encodes a putative single-strand DNA binding protein and T4-like endonuclease VII packaging and recombination enzyme, each of which have been shown to interact with Gp17 during T4 genome packaging.

### Inter and intra-genomic gene flux

Four new insertion sequence (IS) elements, IS*Caje1-4*, were identified in the genomes of these two phages, all belonging to the IS*200*/IS*605 *family. In total five separate IS elements were found in the two genomes, four of which were closely related to each other and belong to the subgroup that encode only TnpB. IS*Caje1 *was found in both CP220 and CPt10, but the two isoforms, which share 97% DNA identity, have inserted into different locations in each genome. IS*Caje2 *in CPt10 and IS*Caje3 *in CP220 show similarity to each other, but are sufficiently different to constitute discrete IS elements (less than 95% identity at the DNA level). Although they were found to insert at the same target sequence (ATTTT), their relative genomic locations were different. The presence of related IS elements in both phage genomes strongly suggests that these phages are derived from the same lineage. They predate upon the same range of hosts, thus allowing the transfer of related insertion elements to occur between phages or between host and phage genomes. The fourth novel IS element, IS*Caje4*, represents a unique insertion in the CP220 genome, and encodes a putative TnpA subunit in addition to TnpB (subgroup IS*608*).

Whether these insertion elements are active during infection is unknown, but they may provide mechanisms whereby regions of the phage genomes can be mobilised, perhaps allowing integration of DNA sequences into the host chromosome or conversely the integration of novel genes into the phage genomes. The presence of putative homing endonucleases at similar sites in both genomes may also provide a partial exclusion mechanism during infection of multiple phage types, or provide a means by which transfer of those genes surrounding the endonuclease can occur, predominantly in favour of the homing endonuclease carrying phage [32,33].

Genetic transfer between host and virulent phage genomes can occur; illegitimate recombination allows the integration of host DNA and if the integrated fragments carry genes that are useful or advantageous to the phage, or to hosts that the phage subsequently infect, they may be maintained in the genome. There is certainly evidence that genes from diverse sources have been integrated into both CP220 and CPt10. Fifteen CDSs in CP220 show significant homology to *Campylobacter *and its taxonomic relatives *Helicobacter*, *Arcobacter *and *Wolinella *(Table 3). The presence of such DNA provides an indication of the importance of phage in the transfer and dissemination of genes between related species as, presumably, if these genes can mobilise onto the phage genome the reverse could also be true. Although during the course of these experiments the gene contents have remained fixed.

**Table 3:** Putative CP220 CDSs showing homology to proteins in *Campylobacter *and closely related species *Helicobacter*, *Arcobacter *and *Wolinella*

CP220 CDS	CP220 Length^a^	Source Organism, Function^b^ Length^a^, Identity (%) (E value)^c^
CPT_0017	264	*Arcobacter butzleri *RM4018.; Radical SAM domain protein 254, 115/252 (45%) (2e^-53^)

CPT_0040	70	*Helicobacter hepaticus *ATCC 51449,; Hypothetical protein HH0764 62, 22/57 (38%) (4e^-04^)

CPT_0066	328	*Campylobacter upsaliensis *RM3195.; Hypothetical protein CUPA0063 305, 83/296 (28%) (2e^-17^)

CPT_0084	342	*Campylobacter fetus *subsp. fetus 82-40.; Heme biosynthesis protein, putative 354, 45/178 (25%) (1e^-07^)

CPT_0095	144	*Campylobacter jejuni *RM1221,; Putative lipoprotein 125, 45/120 (37%) (2e^-12^)

CPT_0105	202	*Campylobacter curvus *525.92.; hypothetical protein CCV52592_0059 189, 109/200 (54%) (1e^-51^)

CPT_0123	143	*Helicobacter hepaticus *ATCC 51449.; Starvation-inducible DNA-binding protein Dps 156, 42/140 (30%) (4e^-12^)

CPT_0127	205	*Campylobacter lari *RM2100.; Hypothetical protein CLA1217 211, 66/109 (60%) (2e^-32^)

CPT_0137	306	*Wolinella succinogenes *DSM 1740.; Molybdenum cofactor biosynthesis protein A 321, 33/98 (33%) (0.002)

CPT_0145	80	*Helicobacter pylori*.; Putative transposase OrfA 210, 31/69 (44%) (3e^-09^)

CPT_0158	182	*Campylobacter upsaliensis *RM3195.; Thymidine kinase 167, 54/170 (31%) (3e^-15^)

CPT_0164	181	*Campylobacter fetus *subsp. fetus 82-40.; Polysaccharide deacetylase 305, 40/151 (26%) (0.006)

CPT_0165	212	*Campylobacter fetus *subsp. fetus 82-40.; FAD-dependent thymidylate synthase 206, 113/200 (56%) (4e^-52^)

CPT_0172	955	*Campylobacter jejuni *RM1221.; Hypothetical protein CJE0595 426, 71/176 (40%) (3e^-32^)

CPT_0191	171	*Helicobacter hepaticus *ATCC 51449.; Hypothetical protein HH0767 188, 61/187 (32%) (6e^-15^)

^a ^Length refers to the number of amino-acid residues in the CDS and are calculated from the genomic sequence

^b ^Gene product and putative function

^c ^Identity and E values are from NCBI BLAST results, performed on 5^th ^January 2009

The products of other CDSs encoded by these phages showed the highest level of sequence identity with those from *Clostridium *sp., *Bacillus *sp., *Porphyromonas *sp. and *Fusobacterium *sp. Thus, the host range of this phage may not have been limited to *C. jejuni *and *C. coli *but extended to other genera. Consistent with this notion, these organisms do share common niches with various *Campylobacter *sp. and therefore provide opportunities for encounters between phage and bacteria to occur.

Rohwer has estimated that there are as many as 2 billion phage associated open reading frames still to be discovered and potentially 100 million different phage genotypes [34]. However, with the availability of an expanded portfolio of phage genome sequences it has become evident that while there are undoubtedly a large number of unique phage genomes, there are many other cases where phages can be clustered into closely related phylogenetic groups [3,35]. That these two phages have maintained their genomes in an almost identical configuration and at such a high level of sequence conservation, despite their independent isolation 14 years apart, suggests that they are well adapted to their hosts; we presume that this is *Campylobacter*, and that just as host bacterial genomes are under selection by their environment there are also significant selective pressures to maintain optimal phage genome structure.

### Distribution of CP220 and CPt10 CDSs in other virulent phage targeting *Campylobacter*

To explore the possibility that these two bacteriophages were part of a conserved lineage targeting *Campylobacter*, we compared a *Campylobacter *phage (CP8) that we had partially sequenced years previously. CP8 had been isolated from chickens independently of both CP220 and CPt10 but unlike the latter had proved refractory to further molecular analysis [10]. Nevertheless CP8 exhibits significant sequence identity with CP220, ranging from 56% up to 100% over a total aggregate sequence length of 11470 bp. We have also extended the current analysis by designing primers for specific regions within CP220 and CPt10 that feature insertion sequences, methylases and structural genes. These primer sets have been used in PCR experiments with genomic DNA isolated from the remaining 15 phages of the UK *Campylobacter *typing scheme.

Five of the phages did not yield DNA amplicons with any of the primer combinations tested (ϕ1, ϕ4, ϕ7, ϕ12 and ϕ16), either due to gene absence, sequence divergence or DNA modifications that prevent efficient PCR amplification [36,37]. However, the remaining 10 phages, of disparate origin, share sequence loci with CP220 and CPt10 (Additional file 3). The typing phages have previously been classified on the basis of genome size by Sails *et al *[17]. Based on this classification CP220 and CPt10 fall within group II, and it is clear from Additional file 3 that the majority of the phages that contain amplicon homologues also belong to group II. Phages 8, 14 and 15 display particularly good correlation with the genes present in CP220 and CPt10. However, ϕ6 isolated in the USA and belonging to group III, also shares at least four genes with the CP220/CPt10 family and in this respect is similar to CP8, which is also a group III phage. Therefore, some genes would seem to be more widely spread in the sampled *Campylobacter *phage meta-genome than others.

The late transcription factor identified in both CP220 and CPt10 appears to be present in all of the group II phages tested but only in one of the group III phages. This may be indicative that these smaller phages are indeed different to the group II class, and have different regulatory factors controlling gene expression. The resolution of these questions requires that we sequence more genomes from these phages.

## Conclusions

We have compared two novel genome sequences from independently isolated virulent *Campylobacter *phages and found them to be extremely similar to each other. Examination of further *Campylobacter *phages has revealed positive matches between a number of them and the two genomes sequenced in this paper, with the implication that the two sequenced phages are well adapted to their particular niche and that there are substantial selective pressures on these phages to maintain this particular genome configuration. It would suggest that phage genomes are as susceptible to selection for stability as they are for variability. Genome stability could be aided by the ability of these phages to display genotypic and phenotypic microvariation by harboring variable length DNA repeat sequences and expressing more than one form of structural protein. Concerted sequencing efforts may reveal the fate of these individual phage lineages and elucidate the extent of genetic flux within *Campylobacter *bacteriophages and between organisms sharing similar niches. However, for most organisms and certainly for *Campylobacter*, the number of sequenced phages is insufficient to ascertain how genome stability would play a role in competition with other phage that may have adopted alternative evolutionary strategies.

## Methods

### *Campylobacter *and Bacteriophage Storage and Growth Conditions

*Campylobacter*strains were stored at -80°C in nutrient broth No. 2 (Oxoid, Basingstoke, UK) supplemented with 20% glycerol or Microbank vials (Prolab Diagnostics, Neston, United Kingdom). Strains were grown on blood agar base No. 2 (Oxoid) or Mueller-Hinton agar (Oxoid), supplemented with 5% defibrinated horse blood (Oxoid), for 24-48 h under microaerobic conditions at 42°C.

Bacteriophage were propagated using the whole plate lysis method, enumerated on *C. jejuni *strains HPC5 (CP220) or NCTC12668 (CPt10) and subsequently stored at 4°C in SM buffer as described previously [10].

### Bacteriophage Purification

Bacteriophages were purified using caesium chloride equilibrium gradients [38]. The suspension was centrifuged at 264,000 *g *in a Beckman TLA 100.3 rotor at 4°C for 24 hours. Residual caesium chloride was removed using a Microcon 30,000 Da cut off column (Millipore, Watford, UK).

### Genome sequencing and analysis

For CP220 a whole genome shotgun library was generated from the purified phage DNA. A shotgun sequencing approach was employed using subclone libraries of size fractionated (inserts 1.4-2 kb and 2-4 kb) phage DNA cloned into vectors pSMART and pUC19. The sequence was assembled from 2138 good quality reads (93% pass rate) that were assembled using Phrap http://www.phrap.org into 3 contigs. A further 290 finishing reads were required to close the gaps in contigs and span low coverage regions by re-sequencing existing library clones or by oligo walks. The finished sequence, a 177493 bp contig consisting of 2376 reads, has a 10-fold read coverage. The CPt10 genome was sequenced by 454 FLX pyrosequencing and assembled from 34265 sequence reads with an average read length of 173 bps and constituting a theoretical 34-fold coverage, using the 454/Roche Newbler assembly program. The gaps between these contigs were closed by directed PCR and the products sequenced with BigDye terminator chemistry on ABI3730 capillary sequencers.

Genome annotation was performed as previously described [39] using Artemis [40]. Insertion sequences were classified using the ISfinder database [41]. The genomes of CP220 and CPt10 were compared pair-wise using the Artemis Comparison Tool (ACT) [42].

### SDS-polyacrylamide gel electrophoresis (SDS-PAGE) and proteomics

Purified phage at log_10 _10 PFU ml^-1 ^were prepared for loading to a 4-15% gradient Tris-HCl polyacrylamide Ready Gel (Bio-Rad, Hemel Hempstead, UK) or 4-12% Bis-Tris NuPAGE Novex gel (Invitrogen, Paisley, UK), using the SDS sample and gel running buffers supplied, according to the manufacturers' instructions. Gels were run at constant voltage of 200 v for 35-50 min followed by staining, with colloidal coomassie blue, according to the manufacturers' instructions. Protein bands were excised from the gel, using a sterile scalpel, for mass spectroscopy analysis.

The gel slices were digested with trypsin, before undergoing electrospray ionization, with subsequent tandem MS/MS. The peptide fragment ions generated were analyzed using Mascot Daemon software [43] or MaxEnt3 maximum entropy software (Waters, Milford, USA).

### Repeat region PCR

Primers used in the amplification of CP220 repeat sequences are shown in Additional file 4. The CP220 genomic sequence was used to design primers in unique flanking regions for each repeat region.

### PCR screening of Campylobacter typing phage

Primers were designed using the CP220 and CPt10 genome sequences to amplify genes of interest including; insertion sequences, methylases and structural genes. Primer sequences are shown in Additional file 5 along with an indication of which genome sequence was used for the design of each primer pair using Primer3 [44].

## Authors' contributions

ART drafted the manuscript. JCY and NKP contributed to drafting the manuscript. ART, JCY, AES and NKP participated in the analysis of the genome sequences. PLC performed the SDS-PAGE analysis. NC performed the repeat sequence experiments. LC, KS and MQ performed the genome sequencing. NRT, DJM and IFC conceived of the study and participated in its design and coordination and critically reviewed the manuscript. All authors read and approved the final manuscript.

## Supplementary Material

Additional file 1 **Supplementary Data - Table S1 - List of CDSs identified in phage CP220 and CPt10**. Table S2 - Repeat region alignments in CP220 and CPt10. Click here for file

Additional file 2 **CP220, CPt10, *Campylobacter jejuni *and *Campylobacter coli *comparative codon usage**. Click here for file

Additional file 3 **Distribution of CP220 and CPt10 CDSs in other phages of the UK *Campylobacter *typing scheme**. Click here for file

Additional file 4 **Primers used in the analysis of CP220 repeat regions**. Click here for file

Additional file 5 **Primers used in the comparative analysis of *Campylobacter *typing phages**. Click here for file
