# Supplementary material for: Evidence for a lineage of virulent bacteriophages that target Campylobacter
Source: BMC Genomics. 2010 Mar 30;11:214. doi: 10.1186/1471-2164-11-214 (PMC2853527; doi:10.1186/1471-2164-11-214)
Supplement: Additional file 2 — CP220, CPt10, Campylobacter jejuni and Campylobacter coli comparative codon usage. [file 1471-2164-11-214-S2.DOC]

**CP220, CPt10, *Campylobacter jejuni* and *Campylobacter coli* comparative codon usage**

|  |  | Cumulative frequency (per 10^3^) | | | |
| --- | --- | --- | --- | --- | --- |
| Amino Acid | Codon | *C.jejuni*^a^ | *C.coli*^b^ | CP220^c^ | CPt10^c^ |
| Arg | CGU | 6.1 | 3.9 | 3.3 | 3.0 |
|  | CGC | 3.6 | 3.3 | 1.1 | 1.0 |
|  | CGA | 0.9 | 1.9 | 1.3 | 1.0 |
|  | CGG | 0.1 | 0.6 | 0.2 | 0.2 |
|  | AGA^d^ | 16.4 | 26.7 | 23.2 | 22.7 |
|  | AGG | 2.7 | 3.1 | 2.1 | 2.4 |
| Leu | UUA | 50.0 | 42.1 | 54.5 | 55.0 |
|  | UUG | 15.3 | 12.3 | 9.9 | 9.7 |
|  | CUU | 31.1 | 21.5 | 12.0 | 12.0 |
|  | CUC | 2.7 | 3.1 | 2.1 | 2.3 |
|  | CUA | 7.0 | 8.2 | 7.5 | 7.1 |
|  | CUG | 1.0 | 1.6 | 1.5 | 1.4 |
| Ser | UCU | 16.1 | 16.1 | 25.3 | 24.2 |
|  | UCC | 2.0 | 2.5 | 2.1 | 2.4 |
|  | UCA | 10.0 | 14.0 | 16.3 | 16.5 |
|  | UCG | 1.5 | 2.0 | 3.0 | 2.7 |
|  | AGU | 22.3 | 16.9 | 18.2 | 17.6 |
|  | AGC | 12.3 | 14.8 | 5.7 | 5.6 |
| Ala | GCU | 33.0 | 34.2 | 25.3 | 24.3 |
|  | GCC | 6.4 | 8.2 | 2.6 | 2.9 |
|  | GCA | 22.1 | 26.1 | 14.2 | 14.8 |
|  | GCG | 5.1 | 4.9 | 2.1 | 2.3 |
| Gly | GGU | 20.1 | 18.5 | 26.1 | 26.6 |
|  | GGC | 8.5 | 11.0 | 4.9 | 4.7 |
|  | GGA | 20.6 | 18.8 | 10.9 | 10.7 |
|  | GGG | 5.7 | 4.9 | 3.2 | 3.4 |
| Pro | CCU | 15.3 | 17.8 | 10.7 | 10.8 |
|  | CCC | 1.3 | 1.9 | 1.2 | 1.2 |
|  | CCA | 8.7 | 9.0 | 14.8 | 15.0 |
|  | CCG | 1.1 | 2.5 | 1.4 | 1.6 |
| Thr | ACU | 18.8 | 22.7 | 27.3 | 27.0 |
|  | ACC | 6.1 | 8.6 | 5.2 | 4.7 |
|  | ACA | 13.4 | 21.3 | 22.0 | 22.4 |
|  | ACG | 2.6 | 2.6 | 2.5 | 2.2 |
| Val | GUU | 22.7 | 24.1 | 27.1 | 27.6 |
|  | GUC | 2.5 | 3.6 | 4.2 | 4.2 |
|  | GUA | 16.9 | 19.9 | 17.0 | 17.5 |
|  | GUG | 9.6 | 8.0 | 4.1 | 3.6 |
| Ile | AUU | 43.7 | 37.2 | 40.1 | 40.9 |
|  | AUC | 16.2 | 16.6 | 10.0 | 10.0 |
|  | AUA | 26.7 | 25.5 | 36.7 | 36.1 |
| Asn | AAU | 55.0 | 54.9 | 64.4 | 64.7 |
|  | AAC | 9.1 | 12.6 | 15.7 | 15.2 |
| Asp | GAU | 48.5 | 45.4 | 58.5 | 58.2 |
|  | GAC | 4.4 | 6.6 | 10.0 | 9.9 |
| Cys | UGU | 8.4 | 5.2 | 12.2 | 11.7 |
|  | UGC | 3.7 | 4.5 | 2.8 | 2.6 |
| Gln | CAA | 28.8 | 31.8 | 27.0 | 27.3 |
|  | CAG | 2.9 | 4.9 | 2.4 | 2.2 |
| Glu | GAA | 57.7 | 52.3 | 57.2 | 57.2 |
|  | GAG | 12.9 | 11.8 | 7.3 | 7.6 |
| His | CAU | 13.0 | 8.2 | 9.3 | 9.1 |
|  | CAC | 3.2 | 3.8 | 4.4 | 4.7 |
| Lys | AAA | 82.6 | 80.6 | 81.3 | 81.9 |
|  | AAG | 13.1 | 12.0 | 13.7 | 13.7 |
| Phe | UUU | 55.3 | 48.0 | 40.2 | 41.5 |
|  | UUC | 4.4 | 7.1 | 11.1 | 11.0 |
| Tyr | UAU | 32.1 | 27.1 | 41.9 | 41.9 |
|  | UAC^d^ | 5.2 | 7.5 | 10.1 | 10.0 |
| Met | AUG | 21.7 | 20.8 | 17.0 | 17.5 |
| Trp | UGG | 6.6 | 8.1 | 7.0 | 7.1 |

^a^ Combined data from three sequenced *C.jejuni* strains; NCTC 11168, RM12221 and 81-176 with a combined total of 1689136 codons. Data is taken from *The Codon Usage Database* [45].

^b^ A partially sequenced *C.coli* strain; RM2228 with 38879 codons. Data is taken from *The Codon Usage Database* [45].

^c^ Codon usage of the two sequenced genomes, only CDSs with recognized start and stop codons were included in the analysis; 51497 codons from CP220 and 48862 codons from CPt10.

^d^ CP220 and CPt10 carry putative tRNAs that recognize these codons.
