# Supplementary material for: Evidence for a lineage of virulent bacteriophages that target Campylobacter
Source: BMC Genomics. 2010 Mar 30;11:214. doi: 10.1186/1471-2164-11-214 (PMC2853527; doi:10.1186/1471-2164-11-214)
Supplement: Additional file 3 — Distribution of CP220 and CPt10 CDSs in other phages of the UK Campylobacter typing scheme. [file 1471-2164-11-214-S3.DOC]

**Distribution of CP220 and CPt10 CDSs in other phages of the UK *Campylobacter* typing scheme**

| Phage Designation | φ2 | φ3 | φ5 | φ6 | φ8 | ϕ9 | φ 11 | φ 13 | φ 14 | φ 15 | CPt10^a^ | CP220 |
| --- | --- | --- | --- | --- | --- | --- | --- | --- | --- | --- | --- | --- |
| Origin^b^ | USA | UK | UK | USA | UK | UK | USA | UK | UK | UK | UK | UK |
| Group^c^ | III | II | III | III | II | III | III | III | II | II | II | II |
| CDS - Structural / Packaging |  |  |  |  |  |  |  |  |  |  |  |  |
| Major capsid protein gp23  (CPT_0051 , CPt10_0491) |  |  |  |  | + |  |  |  | + | + | + | + |
| Portal vertex protein of head  (CPT_0031, CPt10_0291) |  |  |  |  | + |  |  |  | + | + | + | + |
| Baseplate wedge protein  (CPT_0041, CPt10_0391) |  | + |  |  | + |  |  |  | + | + | + | + |
| Tail sheath protein gp18  (CPT_0053, CPt10_0511) |  | + |  |  | + |  |  |  | + | + | + | + |
| Tail tube protein gp19  (CPT_0045, CPt10_0431) |  | + |  |  | + |  |  |  | + | + | + | + |
| Probable phage terminase gp17  (CPT_0001, CPt10_0001) |  | + | + | + | + |  |  |  | + | + | + | + |
| CDS - DNA modification |  |  |  |  |  |  |  |  |  |  |  |  |
| Type III Modification enzyme  (CPt10_0091) |  | + |  | + | + |  | + |  | + | + | + |  |
| Probable DNA methylase  (CPt10_1471) |  |  |  |  | + |  |  |  |  |  | + |  |
| CDS - Insertion elements |  |  |  |  |  |  |  |  |  |  |  |  |
| CP220 and CPt10  (CPT_0138, CPt10_1541) |  |  |  |  | + |  |  |  | + |  | + | + |
| CPT_0151 |  |  |  |  | + |  |  |  | + |  |  | + |
| Hef like homing endonuclease  (CPT_0054c, CPt10_0521) |  |  |  |  |  |  |  |  |  | + | + | + |
| CDS - DNA replication |  |  |  |  |  |  |  |  |  |  |  |  |
| DNA polymerase gp43  (CPT_0115, CPt10_1211) |  |  |  |  | + |  | + |  | + | + | + | + |
| DNA primase-helicase  (CPT_0125, CPt10_1321) |  | + |  |  | + | + |  |  | + | + | + | + |
| DNA topoisomerase  (CPT_0005, CPt10_0041) |  | + |  | + | + |  |  |  | + | + | + | + |
| Sliding clamp loader protein  (CPT_0010, CPt10_0101) |  |  |  |  | + |  |  |  | + | + | + | + |
| DNA ligase  (CPT_0033, CPt10_0321) |  | + |  |  | + |  |  |  | + | + | + | + |
| CDS - Radical SAM |  |  |  |  |  |  |  |  |  |  |  |  |
| CPT_0080 |  |  |  | + |  |  |  |  |  |  |  | + |
| CPT_0083 |  |  |  |  |  |  |  |  |  |  |  | + |
| CPt10_0871 | + |  |  |  | + |  |  |  | + | + | + |  |
| CPt10_0931 |  |  |  |  |  |  |  |  |  |  | + |  |
| CDS - Regulatory |  |  |  |  |  |  |  |  |  |  |  |  |
| Possible transcription factor  (CPT_0048, CPt10_0461) |  | + |  |  | + |  |  | + | + | + | + | + |

+ Indicates a PCR product was obtained using the primers listed in Additional file 5

^a^ CPt10 is φ10 within the UK *Campylobacter* typing scheme

^b^ Phage were originally isolated in the UK or the USA

^c^ Group assignment is based on the classification of Sails *et al* [17] and is according to genome size; group II 180-190kbp and group III 130-140
