# Supplementary material for: Evidence for a lineage of virulent bacteriophages that target Campylobacter
Source: BMC Genomics. 2010 Mar 30;11:214. doi: 10.1186/1471-2164-11-214 (PMC2853527; doi:10.1186/1471-2164-11-214)
Supplement: Additional file 4 — Primers used in the analysis of CP220 repeat regions. [file 1471-2164-11-214-S4.DOC]

**Primers used in the analysis of CP220 repeat regions**

| Repeat Region (CP220^a^) | Primer | Sequence 5’-3’ |
| --- | --- | --- |
| RR1 | CP220-RR1-FP1  CP220-RR1-RP1 | GTTACCAATACTTTAAAGGATGC  AGGATATTCGGTGTGTTCATTC |
| RR3+4 | CP220-RR2-FP1  CP220-RR2-R3 | TTAAAGATAC GCGAACCTGG C  CAACAATGATGAAAGCAATGGC |
| RR6 | CP220-RR3-FP1  CP220-RR3-RP1 | ACCGATGTTATCACAGTTTCAG  GGTTCACCTGGGTATTCGTC |
| RR7 | CP220-RR9-Frd  CP220-RR9-R2 | CAATTCTAGCTATACTGGAAATG  ATGTATCTTTAATAGCTTCGAGC |
| RR8 | CP220-RR4-F3  CP220-RR4-Rev3 | AGCTTTAGATCCTGTGTATTTAG  TAATATGAGATTTCAAGCCCTTG |
| RR9 | CP220-RR5-FP1  CP220-RR4-RP1 | ATTTTTCAAGGGCTTGAAATCTC  GGTTCAGACTAAACCCTGGC |
| RRx | CP220-RR6-Frd  CP220-RR6-Rev | GACGTAGAAGTTGGTATAGCAG  ACATACTTGAGTTACGGTAGCG |

^a^ Repeat regions as currently annotated in the CP220 genome
