# Supplementary material for: Evidence for a lineage of virulent bacteriophages that target Campylobacter
Source: BMC Genomics. 2010 Mar 30;11:214. doi: 10.1186/1471-2164-11-214 (PMC2853527; doi:10.1186/1471-2164-11-214)
Supplement: Additional file 5 — Primers used in the comparative analysis of Campylobacter typing phages. [file 1471-2164-11-214-S5.DOC]

**Primers used in the comparative analysis of *Campylobacter* typing phages**

| Gene^a^ | Primer | Sequence 5’-3’ |
| --- | --- | --- |
| CPT_0051 | JCY001F  JCY001R | cggtgctgtatctccagttg  tttggaagcttaatggcaca |
| CPt10_0291 | 20F  20R | tgctgaatgttggagtggtt  gcatacctttagcaccagcttt |
| CPt10_0391 | 48F  48R | gatggtggagcttacgaagg  tggtgcatttgttttgatgg |
| CPt10_0511  CPT_0053 | 25F  25R  JCY002F  JCY002R | tcagctccaggtattgacga  ggcgattacacttgctggat  ctgaagccattgttgaagca  gacccctgttaagaccagca |
| CPt10_0431 | 26F  26R | tcaagtactaggtgatttagctcgtc  tgaatctgaaaccgaatcca |
| CPt10_0001 | 17F  17R | gtaccaagttcggacgcatt  cagcaaccacttgaccagaa |
| CPt10_0091 | 7F  7R | tcccaaatattgagagcgatt  atgcctatccatttgcgttt |
| CPt10_1471 | 6F  6R | aacagacccaccatacttcg  tttttgtgttgggtgaacga |
| CPT_0138 | 11F  11R | tgctggagcaaaaatagaagg  gccttggttttcggctactt |
| CPT_0151 | 12F  12R | ggtgaagcatctaagagtggaaa  tctttggctccttgcttcat |
| CPT_0054c | 27F  27R | cgtaacagccgcaaaataca  ttcggttctgtgttctccac |
| CPT_0125 | 39F  39R | ggctcctgcaggtattggta  ggaacttctgctggagcttg |
| CPT_0115 | 51F  51R | agggggctttgtagctgaac  agagcttgcaatcgcttcac |
| CPT_0005 | 47F  47R | ggaatcacgctaaagcaggt  ggacaccaccgtctggtatt |
| CPt10_0101 | 19F  19R | caggaaaaacaagcacagca  tctcttgagaaagcactttgg |
| CPt10_0321 | 21F  21R | aacaccggaaataaccagga  cgctaatcccagaaactttacc |
| CPT_0080 | 3F  3R | tcgatggatctacgcaagaa  ggtgcacaccaacgtgtatt |
| CPT_0083 | 4F  4R | gatttgcgacagatgggtct  tccaagaagttgaaagcaaactc |
| CPt10_0871 | 13F  13R | ggggtgaacccacattgtt  ccaagcttttagatccaacttca |
| CPt10_0931 | 14F  14R | cttgtgcaatatgcccacac  aaacgtttttcccaatcgtg |
| CPt10_0461 | 24F  24R | gagtgaaactcgtatgaaacacg  ctcagaacatttttaagttcagca |

^a^ Primers were designed against either the CP220 or CPt10 genome sequences, prefix CPT and CPt10 correspond to the CP220 and CPt10 genomes respectively, loci numbers as listed in the current genome annotation
